# Supplementary material for: Single-Domain Antibodies That Specifically Recognize Intact Capsids of Multiple Foot-and-Mouth Disease Serotype O Strains
Source: Vaccines (Basel). 2025 May 8;13(5):500. doi: 10.3390/vaccines13050500 (PMC12116120; doi:10.3390/vaccines13050500)
Supplement: Supplementary file 1 [file vaccines-13-00500-s001.zip › vaccines-3616053-supplementary.pdf]

Supplementary materials

Single-domain antibodies that specifically recognize intact capsids of multiple foot-and-mouth disease serotype O strains

Michiel M. Harmsen, Nishi Gupta, Quillan Dijkstra, Sandra van de Water, Marga van Setten and Aldo Dekker

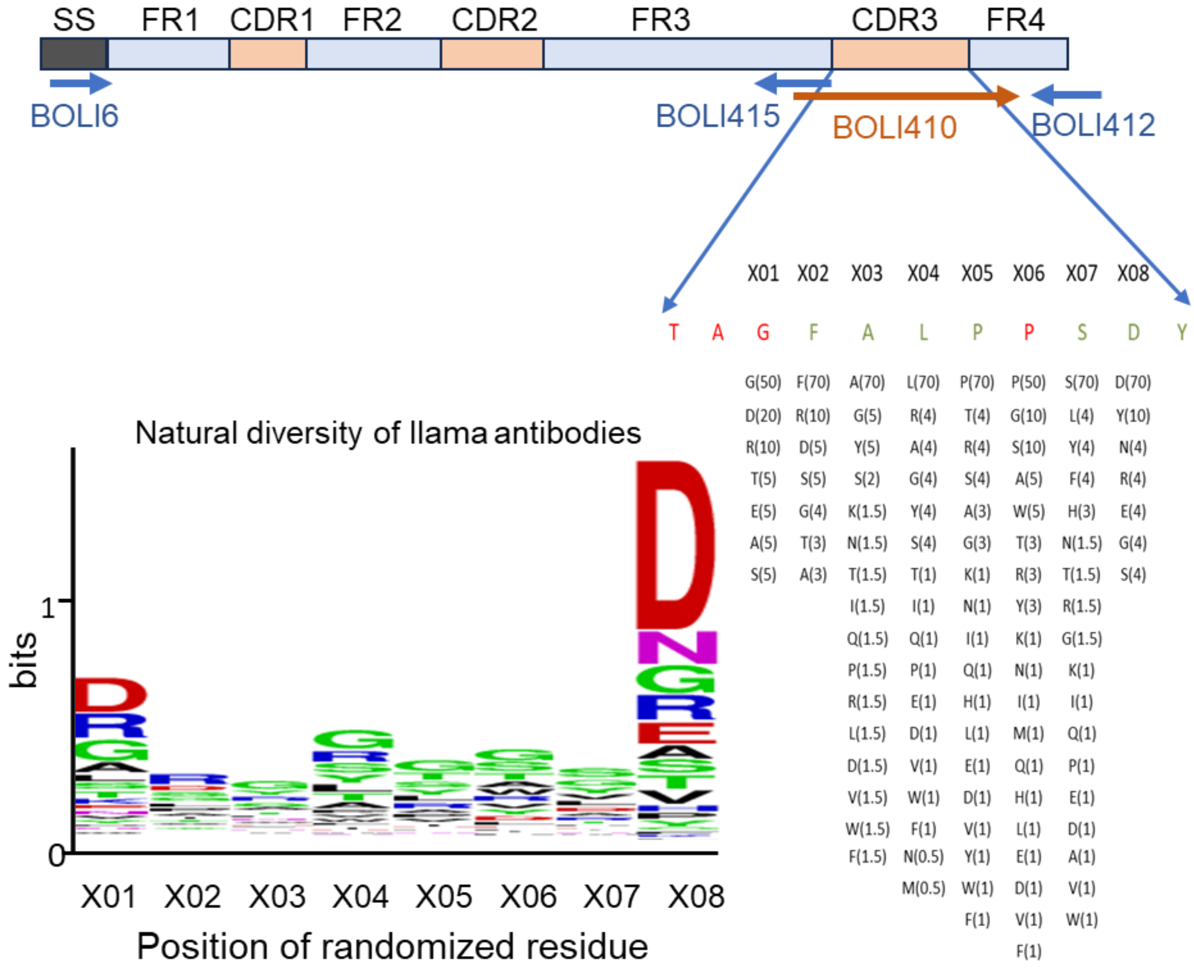

X01	X02	X03	X04	X05	X06	X07	X08
G(50)	F(70)	A(70)	L(70)	P(70)	P(50)	S(70)	D(70)
D(20)	R(10)	G(5)	R(4)	T(4)	G(10)	L(4)	Y(10)
R(10)	D(5)	Y(5)	A(4)	R(4)	S(10)	Y(4)	N(4)
T(5)	S(5)	S(2)	G(4)	S(4)	A(5)	F(4)	R(4)
E(5)	G(4)	K(1.5)	Y(4)	A(3)	W(5)	H(3)	E(4)
A(5)	T(3)	N(1.5)	S(4)	G(3)	T(3)	N(1.5)	G(4)
S(5)	A(3)	T(1.5)	T(1)	K(1)	R(3)	T(1.5)	S(4)
		I(1.5)	I(1)	N(1)	Y(3)	R(1.5)	
		Q(1.5)	Q(1)	I(1)	K(1)	G(1.5)	
		P(1.5)	P(1)	Q(1)	N(1)	K(1)	
		R(1.5)	E(1)	H(1)	I(1)	I(1)	
		L(1.5)	D(1)	L(1)	M(1)	Q(1)	
		D(1.5)	V(1)	E(1)	Q(1)	P(1)	
		V(1.5)	W(1)	D(1)	H(1)	E(1)	
		W(1.5)	F(1)	V(1)	L(1)	D(1)	
		F(1.5)	N(0.5)	Y(1)	E(1)	A(1)	
			M(0.5)	W(1)	D(1)	V(1)	
				F(1)	V(1)	W(1)	
					F(1)		

Figure S1. CDR3 randomization of M170 using a TRIM oligonucleotide approach. The TRIM oligonucleotide BOLI410 ensures mutagenesis of M170 CDR3 by varying the 8 residues at positions X01 to X08 with the amino acid frequencies indicated below the CDR3 sequence. Contact residues of M170 CDR3 with O/BY/CHA/2010 in the cryo EM structure (PDB: 7DST) are indicated in green while non-contact residues are red. The designed amino acid frequency of mutated positions was based on the natural diversity of amino acids at positions X01 to X08 of llama antibodies in the abYsis database, represented by the sequence logo plot. A CDR3-mutagenized M170F DNA fragment was generated by splice overlap extension PCR with four primers and inserted into phage display vector pRL144. BOLI6 localizes on the yeast invertase signal sequence (SS) of vector pRL188 preceding the M170 sequence indicated by FR and CDR regions.

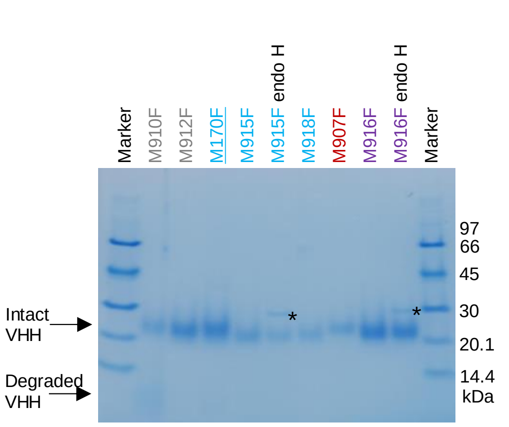

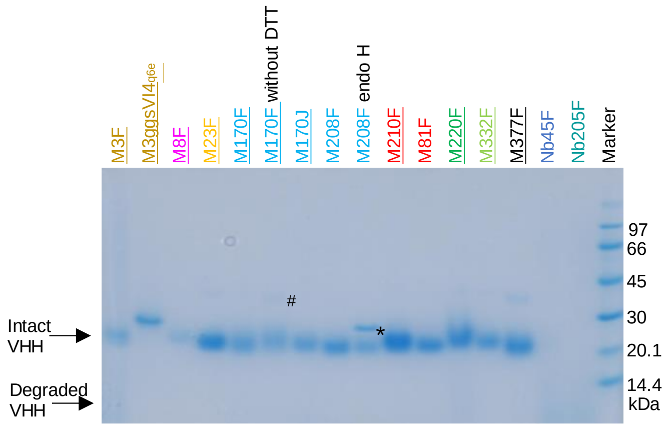

Figure S2. Reducing SDS-PAGE analysis of yeast-produced VHHs. VHHs are color-coded according to CDR3 group. Control VHHs isolated and characterized earlier are underlined. Three VHHs that contain potential a N-glycosylation site were also analysed after treatment with endoglycosidase H (endo H). An asterisk indicates the 30-kDa band representing endo H. A sample of M170F that was analysed without DTT contains an additional band at about 40-kDa (hashtag sign) that represents disulphide-bonded VHH dimers. Some VHHs form a smear below the 14.4-kDa marker, indicating VHH degradation.

Absorbance at 450 nm

	Biotinylated VHH	M3F		M3ggsVI-4$_{Q6E}$		M8F		M23F		M170F		M208F		M915F		M210F		M907F		M912F		M916F		No VHH	
	Coated VHH	146S	12S	146S	12S	146S	12S	146S	12S	146S	12S	146S	12S	146S	12S	146S	12S	146S	12S	146S	12S	146S	12S	146S	12S
O1/Manisa/TUR/69	M3F	2.832	3.533	2.535	3.681	3.357	3.831	2.554	3.834	0.413	**1.661**	0.248	0.186	0.114	0.081	0.142	**1.212**	0.123	0.170	0.068	0.071	0.099	0.106	0.047	0.039
	M3ggsVI-4$_{Q6E}$	2.096	3.143	2.412	3.392	3.474	3.720	2.478	3.533	0.257	**0.519**	0.101	0.154	0.089	0.072	0.131	**1.087**	0.097	0.198	0.049	0.061	0.095	0.104	0.045	0.042
	M8F	3.617	3.825	3.607	3.536	3.357	3.383	3.247	3.219	3.082	**0.693**	2.785	**0.242**	3.309	0.075	2.119	**0.227**	3.199	**0.303**	0.848	0.085	2.886	0.157	0.049	0.049
	M23F	3.828	4.000	3.786	3.860	3.748	4.000	3.594	3.221	3.875	**2.429**	3.581	**0.974**	3.014	0.104	2.773	**0.651**	3.462	**0.401**	1.324	0.100	2.987	0.200	0.054	0.059
	M170F	1.733	**0.291**	2.526	**0.603**	3.689	**0.346**	2.707	0.120	3.114	0.125	2.853	0.071	3.249	0.065	2.044	0.044	2.816	0.056	0.765	0.064	2.514	0.069	0.036	0.046
	M208F	2.179	**0.273**	2.889	**0.602**	3.686	**0.333**	3.060	0.167	3.709	0.082	3.335	0.083	3.355	0.066	2.694	0.057	3.273	0.065	1.283	0.064	2.737	0.067	0.053	0.052
	M915F	2.125	**0.262**	2.344	**0.285**	3.718	**0.287**	2.320	0.104	3.020	0.066	2.935	0.065	2.758	0.061	1.873	0.054	2.762	0.055	0.815	0.056	2.211	0.052	0.049	0.046
	M210F	3.501	**3.257**	3.636	**3.545**	3.818	**3.640**	3.578	**3.262**	3.786	**0.564**	3.388	**0.299**	3.442	0.072	3.105	0.162	3.349	**0.238**	1.111	0.073	3.419	0.123	0.046	0.048
	M907F	1.485	**0.292**	2.519	**0.547**	3.179	**0.397**	1.843	**0.202**	2.626	0.134	2.579	0.160	1.613	**0.201**	1.262	0.066	2.684	**0.244**	0.567	0.134	2.098	0.188	0.051	0.050
	M912F	0.285	0.180	0.321	**0.213**	2.200	**0.244**	0.488	0.099	1.310	0.058	1.339	0.060	1.187	0.050	0.632	0.045	1.997	0.048	0.663	0.048	1.434	0.053	0.025	0.042
	M916F	2.452	**0.384**	2.569	**0.442**	4.000	**0.456**	3.013	0.150	3.880	0.084	3.654	0.064	3.218	0.070	2.981	0.058	3.692	0.064	1.536	0.063	3.195	0.068	0.056	0.050
	No VHH	0.091	0.167	0.108	0.183	0.143	**0.244**	0.068	0.084	0.078	0.050	0.070	0.048	0.068	0.047	0.058	0.048	0.072	0.047	0.049	0.047	0.065	0.047	0.047	0.048
O/SKR/7/2010	M3F	3.122	3.151	2.197	2.951	3.090	3.674	0.106	0.227	0.086	**0.215**	0.071	0.069	0.061	0.069	0.129	**0.471**	0.095	0.107	0.102	0.063	0.126	0.075	0.042	0.044
	M3ggsVI-4$_{Q6E}$	2.865	3.362	2.129	3.233	2.194	3.680	0.093	0.241	0.090	**0.208**	0.065	0.078	0.059	0.060	0.118	**0.415**	0.102	0.099	0.099	0.058	0.137	0.088	0.045	0.047
	M8F	3.628	2.957	2.281	3.098	2.187	2.666	0.171	0.114	0.349	**0.266**	0.542	0.069	0.206	0.059	2.341	0.126	2.040	0.149	2.213	0.063	3.254	0.113	0.049	0.054
	M23F	3.373	3.865	3.076	3.239	3.461	3.308	0.125	0.094	0.367	**0.444**	0.703	0.104	0.244	0.074	3.035	0.191	2.117	0.178	2.578	0.087	2.969	**0.216**	0.057	0.060
	M170F	0.275	0.110	0.385	0.112	1.486	0.139	0.082	0.078	0.116	0.073	0.134	0.077	0.081	0.060	0.513	0.056	0.487	0.053	0.713	0.055	1.104	0.062	0.049	0.054
	M208F	0.653	0.116	0.711	0.113	2.742	0.132	0.085	0.076	0.142	0.070	0.301	0.088	0.120	0.064	1.460	0.057	1.106	0.054	1.497	0.060	1.869	0.070	0.049	0.052
	M915F	0.120	0.075	0.316	0.113	0.058	0.054	0.057	0.049	0.055	0.056	0.135	0.063	0.068	0.051	0.054	0.072	0.354	0.049	0.562	0.044	0.617	0.052	0.045	0.060
	M210F	3.383	**4.000**	3.060	**2.740**	3.701	**2.756**	0.288	0.168	0.543	**0.348**	1.057	0.120	0.263	0.063	4.000	0.104	2.184	0.163	2.572	0.068	3.041	0.145	0.047	0.046
	M907F	0.848	0.176	0.886	0.169	2.978	**0.374**	0.274	0.194	0.241	0.115	0.493	0.152	0.219	0.149	1.915	0.070	1.626	0.143	2.305	0.119	2.841	0.182	0.045	0.051
	M912F	0.425	0.070	1.891	0.113	0.179	0.044	0.272	0.041	0.053	0.048	1.032	0.056	0.191	0.045	0.058	0.062	2.237	0.035	2.385	0.039	2.728	0.050	0.034	0.031
	M916F	2.165	**1.336**	1.892	0.120	0.727	0.078	1.094	0.057	0.063	0.064	4.000	0.073	0.270	0.058	0.058	0.057	2.555	0.064	3.356	0.060	2.883	0.064	0.051	0.051
	No VHH	0.061	0.055	0.064	0.095	0.045	0.045	0.037	0.046	0.046	0.044	0.056	0.053	0.044	0.045	0.047	0.047	0.053	0.046	0.061	0.043	0.067	0.046	0.044	0.046
O/TAW/3/97	M3F	3.317	3.536	2.640	3.317	3.726	3.870	0.489	1.368	0.171	**0.311**	0.103	**0.206**	0.101	0.089	0.106	0.088	0.152	0.184	0.075	0.092	0.117	0.136	0.052	0.061
	M3ggsVI-4$_{Q6E}$	2.910	4.000	2.182	2.955	3.493	3.272	0.372	1.816	0.137	**0.271**	0.099	0.183	0.059	0.077	0.060	0.084	0.105	0.108	0.064	0.080	0.091	0.099	0.047	0.050
	M8F	3.032	3.468	2.704	2.770	3.115	2.021	0.672	0.284	0.299	**0.379**	0.181	**0.276**	0.062	0.068	0.082	0.095	0.391	0.188	0.561	0.074	2.129	0.108	0.054	0.054
	M23F	3.683	4.000	3.631	3.288	3.772	2.995	1.468	0.414	0.670	**0.764**	0.518	**0.601**	0.115	0.115	0.111	0.135	0.946	**0.298**	1.459	0.083	2.768	0.183	0.055	0.054
	M170F	0.107	**0.200**	0.129	0.139	0.278	0.108	0.255	0.168	0.181	0.084	0.078	0.082	0.066	0.103	0.058	0.058	0.099	0.067	0.081	0.067	0.199	0.059	0.047	0.052
	M208F	0.240	**0.324**	0.164	0.177	0.713	**0.347**	0.125	0.198	0.142	0.083	0.086	0.075	0.071	0.069	0.061	0.059	0.146	0.071	0.147	0.080	0.490	0.065	0.048	0.055
	M915F	0.072	0.066	0.120	0.075	0.252	0.074	0.070	0.065	0.060	0.060	0.064	0.064	0.055	0.056	0.055	0.051	0.058	0.054	0.057	0.049	0.135	0.063	0.054	0.072
	M210F	0.262	0.138	0.222	0.127	1.421	**0.232**	0.184	0.093	0.080	0.073	0.095	0.087	0.060	0.060	0.055	0.060	0.177	0.063	0.188	0.061	1.133	0.059	0.049	0.049
	M907F	0.283	0.156	0.313	0.153	2.666	**0.314**	0.317	**0.205**	0.144	0.127	0.184	0.156	0.163	0.182	0.078	0.073	0.286	0.174	0.339	0.133	1.492	0.166	0.054	0.058
	M912F	0.175	0.065	0.425	0.070	2.625	0.070	0.236	0.061	0.061	0.051	0.065	0.064	0.053	0.048	0.045	0.044	0.179	0.044	0.272	0.041	1.032	0.056	0.058	0.062
	M916F	0.843	**0.527**	2.165	**1.336**	3.727	**0.440**	0.899	0.080	0.081	0.095	0.103	0.081	0.063	0.064	0.062	0.060	0.727	0.078	1.094	0.057	4.000	0.073	0.058	0.057
	No VHH	0.054	0.054	0.061	0.055	0.056	0.050	0.046	0.045	0.047	0.046	0.049	0.047	0.046	0.044	0.045	0.047	0.045	0.045	0.037	0.046	0.056	0.053	0.047	0.047

Figure S3. Particle specificity of VHHs in DAS-ELISAs using different VHHs for coating and as biotinylated VHH. BEI-inactivated antigens from three FMDV strains were used in VHH-based DAS-ELISAs at 1 µg/mL either untreated (146S) or after heating at 56 ^{0}C for 1 h (12S). For all three strains a matrix of different combinations of VHHs were used for coating and as biotinylated VHH. VHHs are color coded according to their CDR3 group. Absorbance values are represented using a red-blue color scheme to visualize different values. Absorbance values showing unexpected binding to 12S particles by presumed 146S-specific VHHs are underlined when above 0.1 and also bold when above 0.2.

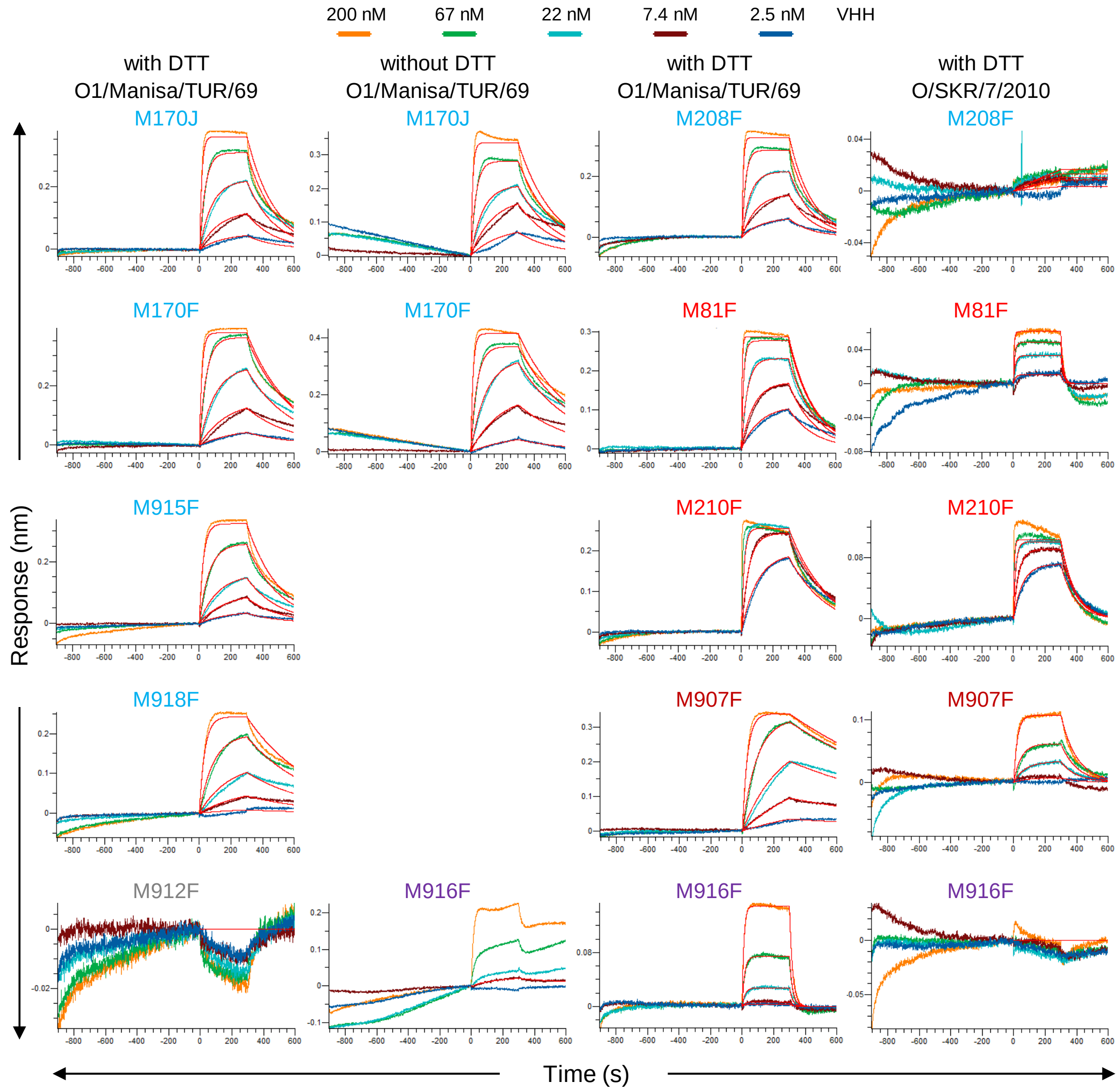

Figure S4. Biolayer interferometry analysis of VHH binding affinity to FMDV 146S particles. Biotinylated 146S-specific VHHs M170F or M210F were coupled to SAX sensors for subsequent capture of O1/Manisa/TUR/69 or O/SKR/7/2010 146S particles, respectively. The graphs show the three subsequent incubations of 146S particle loaded sensors in buffer until t=0 s (baseline), association phase with unlabelled VHH from t=0 s to t=300 s and dissociation phase from t=300 s to t=600 s in buffer. The use of 10 mM DTT in the assay buffer PBST to reduce the VHH analyte as well as the FMDV strain used is indicated above each column of graphs. VHHs are color coded according to their clonal group. Each graph depicts data for one VHH interacting with 146S particles at five different VHH concentrations as indicated by the legend above. These were used for global curve fitting to a 1:1 interaction model to determine the binding affinity. Red lines are the fitted curves. Only for M916F without DTT binding to O1/Manisa/TUR/69 a single VHH concentration was used for curve fitting, due to the strong baseline drift at the other VHH concentrations. M912F binding to O1/Manisa/TUR/69 and M208F and M916F binding to O/SKR/7/2010 could not be detected.
